# Contribution of Gut Microbiota to Immunological Changes in Alzheimer’s Disease

**DOI:** 10.3389/fimmu.2021.683068

**Published:** 2021-05-31

**Authors:** Lynn van Olst, Sigrid J.M. Roks, Alwin Kamermans, Barbara J. H. Verhaar, Anne M. van der Geest, Majon Muller, Wiesje M. van der Flier, Helga E. de Vries

**Affiliations:** ^1^ Department of Molecular Cell Biology and Immunology, Amsterdam University Medical Centers, Amsterdam Neuroscience, Amsterdam, Netherlands; ^2^ Department of Internal Medicine, Section Geriatrics, Amsterdam UMC, Vrije Universiteit Amsterdam, Amsterdam Cardiovascular Sciences, Amsterdam, Netherlands; ^3^ Vrije Universiteit Amsterdam, Athena Institute, Amsterdam, Netherlands; ^4^ Alzheimer Center Amsterdam, Department of Neurology, Amsterdam Neuroscience, Vrije Universiteit Amsterdam, Amsterdam UMC, Amsterdam, Netherlands; ^5^ Department of Medical Biochemistry, Amsterdam Cardiovascular Sciences, University of Amsterdam, Amsterdam, Netherlands

**Keywords:** Alzheimer’s disease, gut microbiota, neuroinflammation, immune cells, therapeutic intervention, microbial metabolites

## Abstract

Emerging evidence suggests that both central and peripheral immunological processes play an important role in the pathogenesis of Alzheimer’s disease (AD), but regulatory mechanisms remain unknown. The gut microbiota and its key metabolites are known to affect neuroinflammation by modulating the activity of peripheral and brain-resident immune cells, yet an overview on how the gut microbiota contribute to immunological alterations in AD is lacking. In this review, we discuss current literature on microbiota composition in AD patients and relevant animal models. Next, we highlight how microbiota and their metabolites may contribute to peripheral and central immunological changes in AD. Finally, we offer a future perspective on the translation of these findings into clinical practice by targeting gut microbiota to modulate inflammation in AD. Since we find that gut microbiota alterations in AD can induce peripheral and central immunological changes *via* the release of microbial metabolites, we propose that modulating their composition may alter ongoing inflammation and could therefore be a promising future strategy to fight progression of AD.

## Introduction

Alzheimer’s disease (AD) is a neurodegenerative disorder of which the prevalence and disease burden are increasing simultaneously with an aging population (1, 2). Extracellular amyloid-beta (Aβ) deposition and intracellular accumulation of hyperphosphorylated tau are the primary neuropathological hallmarks of AD (3), but increasing attention addresses an additional role of distorted immune responses, although underlying mechanisms remain unknown (4, 5).

Gut microbiota are important for peripheral and central immune homeostasis (6). Species of microbiota and their metabolites can induce peripheral immune activation and contribute to a systemic immune response (7). Moreover, they can modulate integrity of the blood-brain barrier (BBB) (8), which regulates migration of immune cells into the brain (5). In addition, the production of certain metabolites by gut microbiota is linked to the maturation and function of microglia, the CNS resident immune cells (9).

Over thousands of microbial taxa are present in the adult gastrointestinal tract (GI) where interaction takes place between the host, microbial antigens, and environmental factors (1, 10). Most of these belong to the gram-negative phylum *Bacteroidetes* (1, 11–15) and the gram-positive phylum *Firmicutes* (1, 11–14, 16) with a smaller proportion of gram-positive *Actinobacteria* (11, 14, 17), and gram-negative *Proteobacteria* (14, 18) and *Veruccomicrobia* (12, 19). Within the two dominant phyla, abundant genera include *Bacteroides*, *Clostridium, Faecalibacterium*, *Roseburia* and *Eubacterium* (11, 12, 14). Alterations in microbiota composition occur with increasing age (7), starting around 65 years and include an increase in *Bacteroidetes* and a decrease in *Firmicutes* (20, 21).

**Figure 1:**
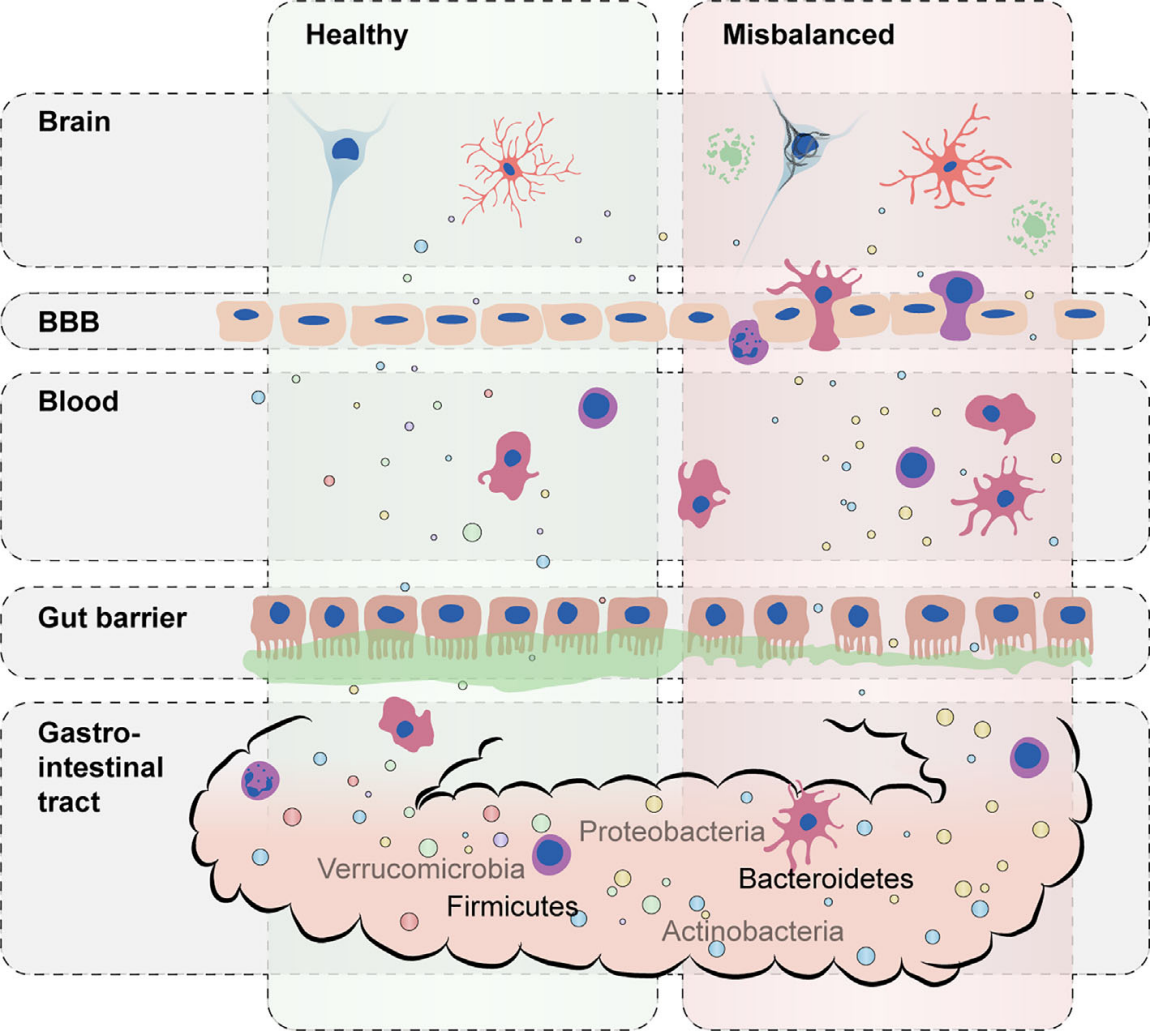
Schematic representation of the impact of a misbalanced gut microbiota on host immunity. A balanced composition of gut microbiota with a high diversity of commensal bacteria carrying out essential microbial functions supports healthy immune responses (left). During microbiota misbalance (right), excessive proinflammatory cytokines and bacterial toxins (e.g., lipopolysaccharide) can lead to disruption of gut permeability and blood–brain barrier (BBB) integrity. Distorted immune responses in the brain can further accelerate and worsen AD-associated pathology such as Aβ and tau accumulation.

Gut microbiota can affect host immunity *via* the release of metabolites and toxins. Microbial misbalance can lead to systemic inflammation in the gut and affect the gut barrier function, increasing permeability and the entry of bacteria, metabolites and toxins into the circulation (22) (**Figure 1**). Interestingly, gut permeability was recently reported to be increased in a cohort of dementia patients together with the occurrence of systemic inflammation (23). Lipopolysaccharide (LPS) is a pro-inflammatory endotoxin found in the outer membrane of gram-negative bacteria like *Bacteroidetes* (15, 24, 25). Besides LPS, some gram-negative species also excrete polysaccharide A (PSA), which has an anti-inflammatory potential (7, 26–28). Other metabolites that predominantly exert immunoregulatory properties are short-chain fatty acids (SCFAs), of which butyrate in particular is known to be produced by species within *Firmicutes*, mainly within clusters of the *Clostridia* class (16, 29–31).

During adult life, microbiota composition can be influenced by different factors such as diet (2, 32, 33), environment (34), body mass index (BMI) (34), cholesterol (34), lifestyle factors such as smoking and exercise (32), drug use (32), and ethnicity (35, 36). As such, microbiota components differ greatly between individuals (37). Despite this variation, a common set of microbial taxa can be found across individuals with diverse dietary habits, geographic origin and ethnicity, referred to as the “core microbiome” (12, 38–40). The core microbiome (11, 12) is essential for microbial functions even if not carried out by the same group of microbes (10) and depends on the expression of specific combinations of microbial genes, together with metabolic processes and regulatory pathways. Hence, higher diversity in microbiota composition is associated with better health (41).

Aging is the most important risk factor for AD. During aging, the gut microbiota composition decreases in diversity and stability (42). It has been postulated that these changes may evoke hyperstimulation of the immune system resulting in persistent, low-grade inflammation (43), referred to as “inflammaging” (4), a phenomenon observed in the elderly. In addition, decreased immune function, or ‘immunosenescence’, is a hallmark of aging. Both inflammaging and immunosenescence contribute to aging of the peripheral immune system, which is associated with higher susceptibility to infection, increased risk of autoimmune diseases and impaired cognitive function (4, 43).

In this literature review, we will discuss gut microbiota alterations in both animal model and human studies of AD. Altered microbial taxa and associated metabolites will be described in relation to changes in immune and BBB function. Finally, we will elaborate on possible strategies to target gut microbiota to restore immune homeostasis in AD.

## Microbiota Composition in AD Patients and AD Animal Models

### Animal Studies

**Figure 2:**
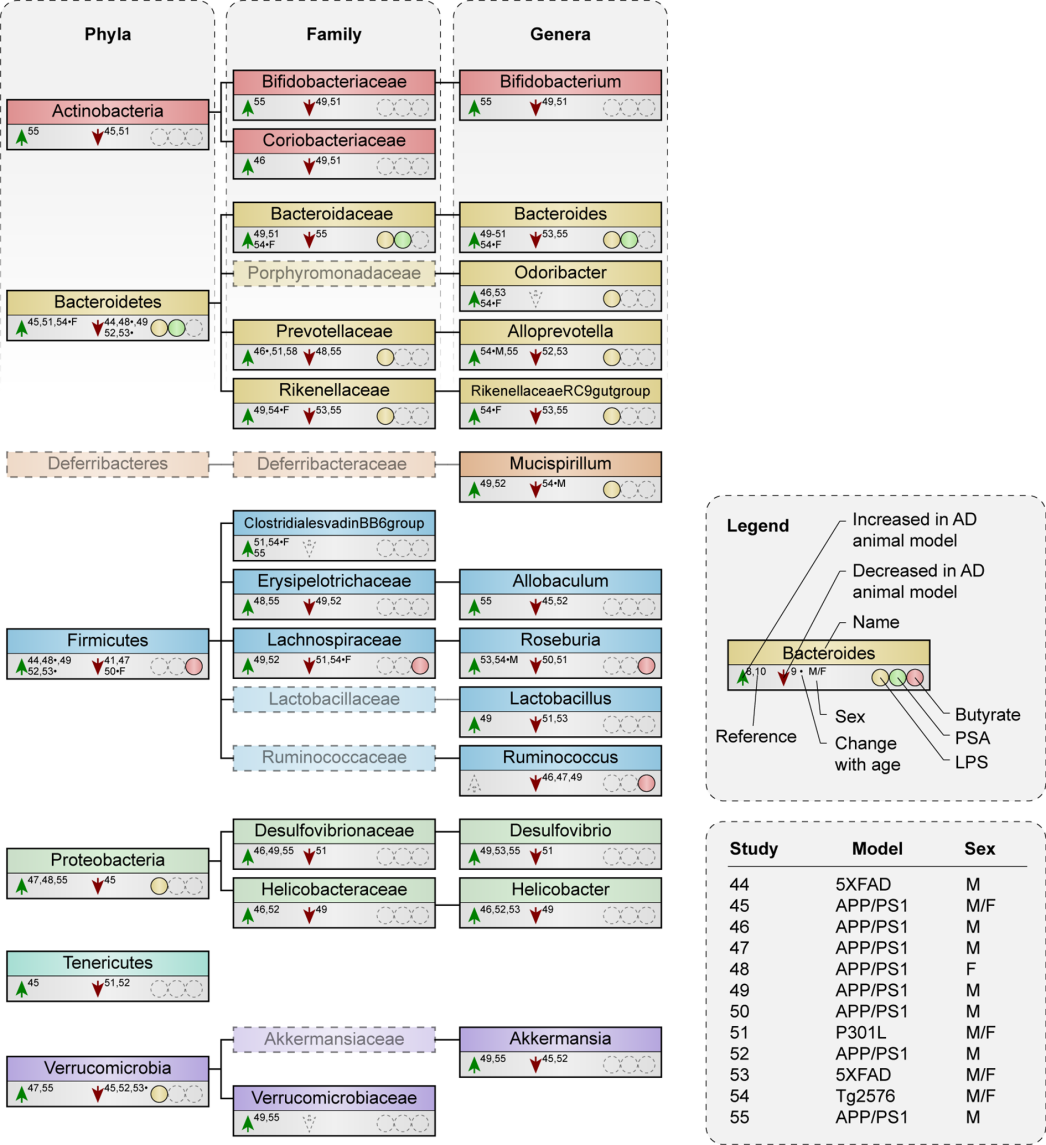
Microbial taxa are altered in AD mouse models compared to WT mice. Phylogenetic representation at phylum, family and genus level of microbial taxa described by three or more animal studies. Animal studies are represented that either compare microbiota composition between AD and WT mice at certain age point(s), or that examine alterations with increasing age in AD mice compared to WT. Arrows indicate an increase or decrease in abundance of a certain taxa in AD mouse models compared to WT. * indicates a result was observed in AD mice with increasing age, but not in WT. F or M show a change that was only seen in females or males respectively, if both were included in one study. Presence or excretion of toxin lipopolysaccharide (LPS; yellow) and metabolites polysaccharide A (PSA; green) and butyrate (red) is indicated, as well as the used animal model and sex of the animals used per study.

Various studies have investigated gut microbiota composition in AD mouse models (**Supplementary Table 1**) (44–55), most in the context of Aβ pathology (44–50, 52–55). Microbial taxa described by three or more studies are shown in **Figure 2**, while **Supplementary Table 2** demonstrates all altered taxa in AD mouse models compared to wild type (WT). All studies observed changes in microbiota composition at one or more taxonomic levels in mouse models of AD compared to WT, although very few alterations were mentioned at species level (only shown in **Supplementary Table 2**).

Studies that investigated changes in microbiota composition during the disease course in animal models found that *Firmicutes* and *Bacteroidetes* abundance both increased and decreased compared to levels in the WT (48, 53, 54). Butyrate producers like the family *Lachnospiraceae* reduced during pathology in AD mice of both sexes while *Roseburia*, a genus within *Lachnospiraceae*, increased over time in male AD mice (54).

Both higher (44, 49, 52) and lower (45, 51) abundance was reported of the phylum *Firmicutes* in AD mouse models. Families and genera that contain butyrate-producing species like the family *Lachnospiraceae* (49, 51, 52) and the genus *Roseburia* (50–52) were also both increased and reduced in AD mice compared to WT. Notably, the butyrate-producing genus *Ruminococcus* was either unchanged or decreased (46, 47, 49). Similar to the abundance of and within *Firmicutes*, higher (45, 51) and lower (44, 49, 52) abundance was reported of the LPS-containing phylum *Bacteroidetes* and of its families and genera (46, 48, 49, 51, 52, 54, 55). Only the genus *Odoribacter* was either unchanged or increased (46, 53).

The different findings regarding microbiome alterations in AD mouse models in the aforementioned studies appear to be independent of age, diet and the type of model, which was either based on Aβ pathology in the APP/PS1 (45–50, 52, 54, 55), 5XFAD (44, 53) and Tg2576 models (54), or on tau pathology in the P301L model (51). Divergent results in microbiota composition can be a result of the use of male or female mice. First, because microbiota composition in male and female mice is under the influence of sex-specific hormones (56, 57) and second, because AD pathology manifest itself differently between sexes. Studies using only male mice (44, 49, 52) showed a reduction in *Bacteroidetes* and an increase in *Firmicutes* in AD mouse models, while studies using both males and females varied in their results (45, 51, 53). At each age, the ratio of *Firmicutes/Bacteroidetes* remained lower in AD than in WT mice in females (48), suggesting that females more often show an increase in *Bacteroidetes* and a reduction in *Firmicutes* as opposite to male AD mice. Gram-negative families such as *Bacteroidaceae* are both increased and reduced in male mice in relation to AD (46, 49, 52, 55), but more often increased in females (48, 54). Families that encompass butyrate-producers like *Lachnospiraceae* (49, 52) show increase in male mice, while studies that included females or both sexes demonstrated a decrease (51, 54). Microbiota alterations in the gram-negative genus *Bacteroides* and butyrate-producing genus *Roseburia* across different studies seem less sex-specific, although one study demonstrated an increase in *Bacteroides* in females specifically, while males show an increase in *Roseburia* (54). Since the large majority of studies use either male mice or a combination of males and females, it is possible that the overall results are more specific for males than they are for females.

Altogether, it seems that various alterations in microbiota composition are associated with pathology and disease progression in AD animal models. This suggests that general microbiota misbalance, rather than alterations in specific taxa, is characteristic for AD.

### Human Studies

So far, only three studies have directly compared gut microbiota composition in AD patients to controls (58–60) and one studied microbiome associations with amyloid pathology (61) (**Supplementary Table 3**).

**Figure 3:**
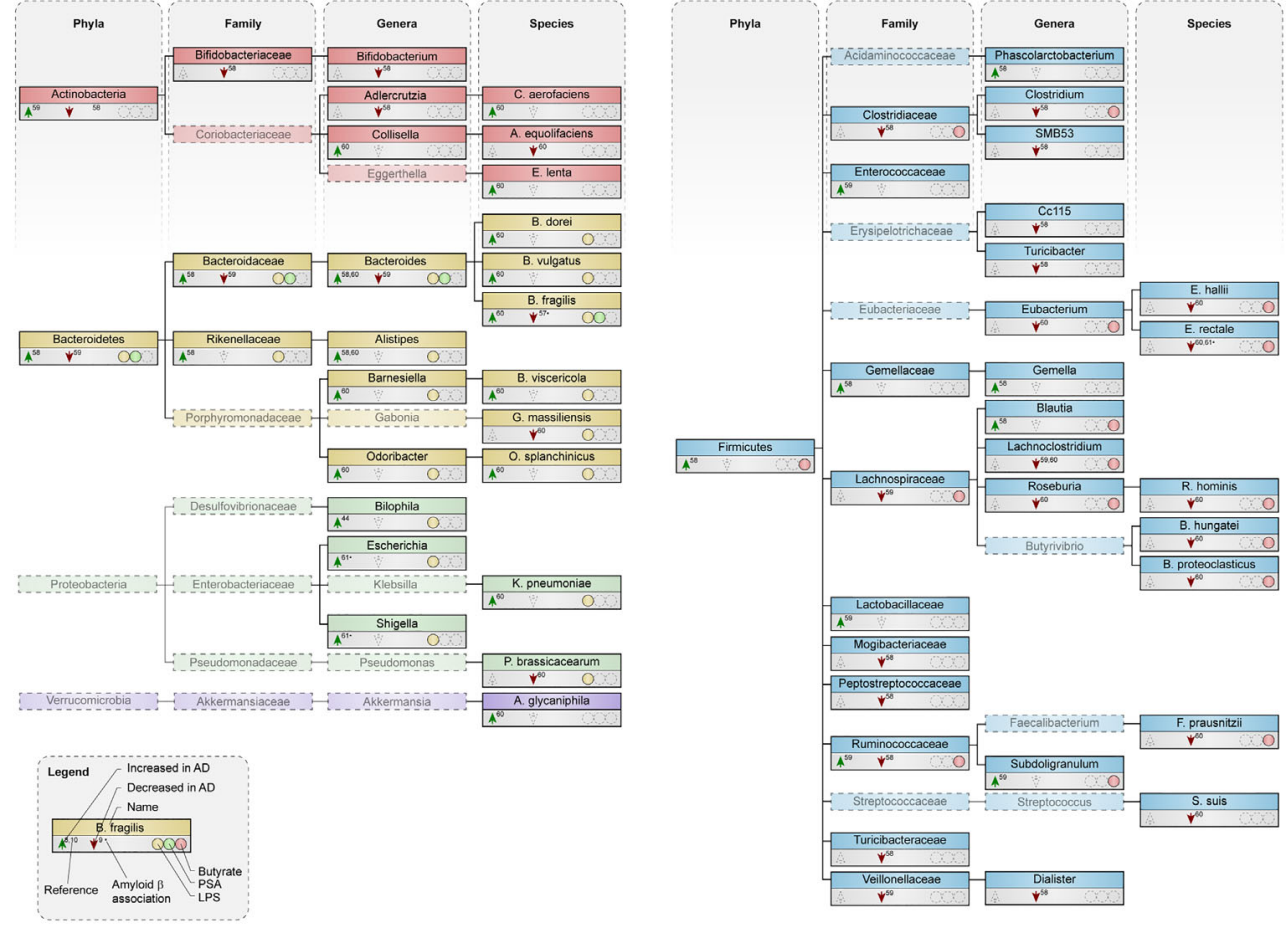
Microbial taxa are altered in AD patients compared to control subjects. Phylogenetic representation at phylum, family, genus and species level of microbial taxa in human studies. Arrows indicate an increase or decrease in abundance of a certain taxa in AD patients compared to healthy controls. * depict changes associated with amyloid pathology in cognitively impaired patients. Presence or excretion of toxin lipopolysaccharide (LPS; yellow) and metabolites polysaccharide A (PSA; green) and butyrate (red) is indicated.

Both an increase (58) and a slight decrease (59) in abundance of the phylum *Bacteroidetes* was reported in AD patients (**Figure 3**). All species within the phylum *Bacteroidetes* are gram-negative and contain the toxin LPS in their outer membrane (15, 24, 25). Higher circulating levels of LPS were associated with increased Aβ deposition in elderly patients with cognitive complaints (62). Increased abundance was detected in most genera within *Bacteroidetes* and most of its species, including *B. fragilis* (60), which produces polysaccharide A (PSA) (7). Interestingly, *B. fragilis* was decreased in cognitively impaired patients that were Aβ-positive compared to healthy subjects (61).

Abundance of the phyla *Firmicutes* was both reduced (58) and unchanged (59) in AD patients. Within *Firmicutes*, a reduction was seen in the family *Lachnospiraceae* (59, 60) the genus *Roseburia* (60) and the genus *Eubacterium* (60), which all harbor species that produce the metabolite butyrate. At species level, butyrate-producers like *R. hominis*, *F. prausnitzii, E. rectale* and *E. hallii* were lower abundant in AD patients (60). Interestingly, butyrate levels in plasma negatively associated with Aβ deposition in cognitively impaired patients (62).

The deposition of Aβ in cognitively impaired patients positively associates with the abundance of the gram-negative genus *Escherichia/Shigella*, while the butyrate-producing species *E. rectale* negatively relates to Aβ deposition in these patients, both as compared to Aβ-negative cognitively impaired patients and healthy controls (61). In addition, differences in variation of microbial taxa within an individual, which is also called α-diversity and associates with better health (41), was decreased in AD patients (58).

Available evidence regarding microbiota composition in patients with AD faces several limitations, including small sample sizes and limited data on and adjustment for dietary intake and other relevant confounding factors, such as co-morbidity, use of medication and lifestyle. Only two of the aforementioned studies (60, 61) reported microbiota composition at species level. Few studies related the observed differences between groups to disease biomarkers and severity, such as cerebral spinal fluid (CSF) or PET biomarkers for Aβ and tau, MRI characteristics or cognitive functioning. Therefore, results should be interpreted with caution. Limited human data points towards higher abundance of gram-negative species containing LPS while species that produce the metabolite butyrate were decreased. Besides, phyla, families and genera encompassing these LPS-containing and butyrate-producing species were also mostly increased and decreased respectively.

## Microbiome-Immune Interactions

**Figure 4:**
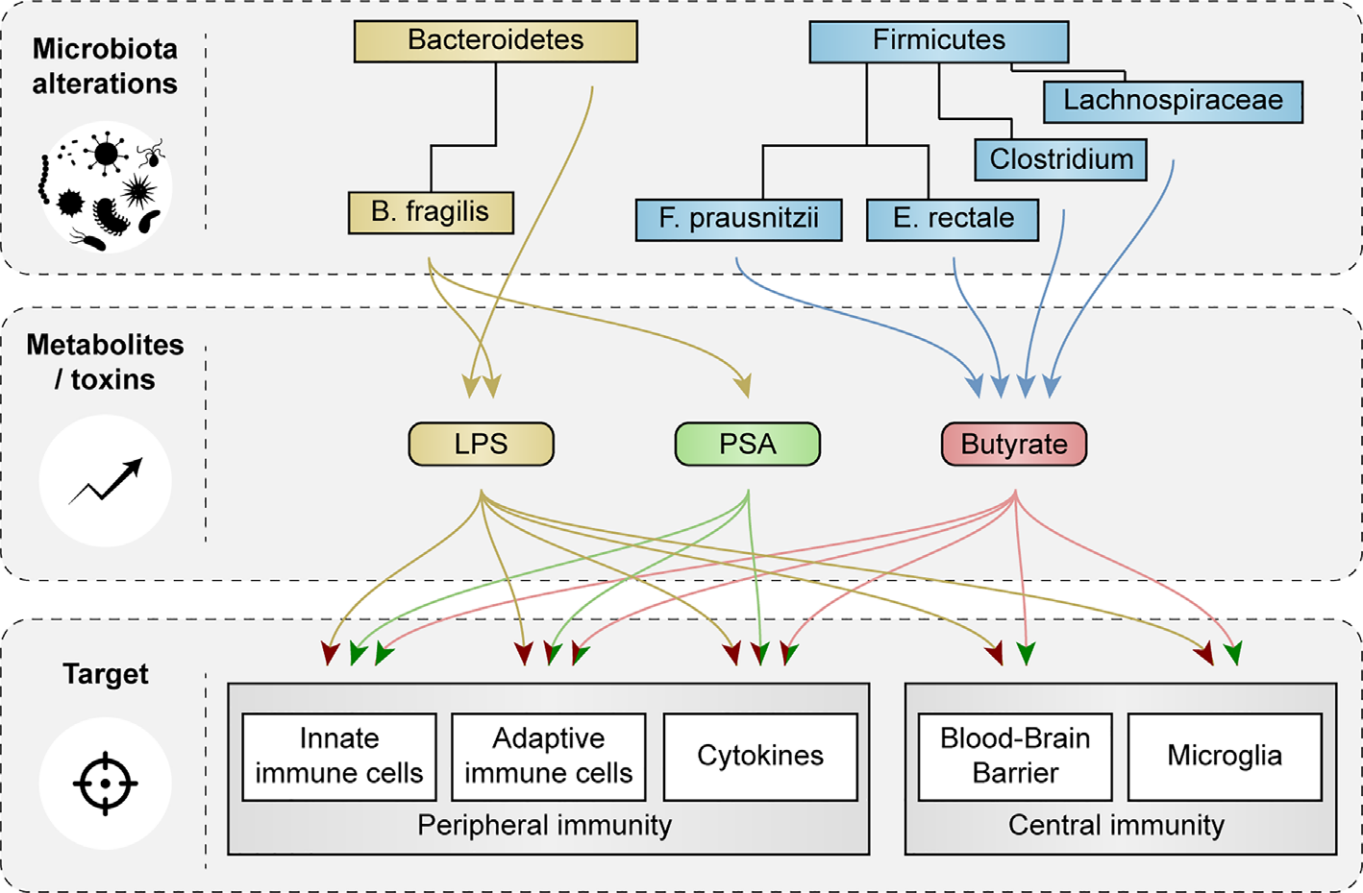
Schematic representation of the effects of microbial metabolites and toxins on peripheral and central immunity and blood-brain barrier function. Impact of microbial metabolites and toxins, that were changed in AD patients and in relevant animal models, on peripheral and central immune cells, cytokine secretion and blood-brain barrier (BBB) function are showed. Red arrow heads indicate a pro-inflammatory effect or loss of BBB integrity, green arrow heads indicate tolerogenic effects and improvements in BBB function.

*Bacteroidetes* and *Firmicutes* make up the largest portion of the adult microbiota and showed most changes in abundance in AD patients and in relevant animal models. Here, we discuss how species of *Bacteroidetes* and *Firmicutes* and their metabolites cause activation or inhibition of peripheral and central immune cells and how they affect function of the BBB (**Figure 4**).

### Gram-Negative Bacteria

Bacteria within the phylum *Bacteroidetes* are gram-negative and contain LPS (15, 24, 25). LPS can induce systemic inflammation *via* Toll-like receptor (TLR)-4 signaling (15, 24, 25) and promotes the secretion of proinflammatory cytokines like interleukin 1 and 6 (IL-1 and IL-6) and tumor necrosis factor α (TNF-α) (63). IL-1 and IL-6 are required for differentiation of T-helper 17 (Th17) cells (64), which *via* proinflammatory cytokine release and their action on neurons *via* the Fas/Fas-ligand apoptotic pathway, are thought to contribute to neuroinflammation and neurodegeneration in AD (65). In addition, LPS of the species *B. fragilis* induced signaling *via* nuclear factor kappa-light-chain-enhancer of activated B cells (NF-κB) in human neuronal-glial co-cultures, an important pathway in inflammatory neurodegeneration (66). Moreover, increased abundance of the genus *Bacteroides* positively associated with cerebrospinal fluid (CSF) levels of chitinase 3 like protein 1 or YKL-40, which is a marker for microglial and astroglial cell activation (58). Increases in species of *Bacteroidetes* might contribute to LPS transport from the intestines to the brain, adding to AD pathology (33, 67). Interestingly, LPS has been found at higher levels in the parenchyma and vessels of AD brains compared to aged-matched controls, and co-localized with Aβ plaques around blood vessels (67).

LPS has also been described to increase P-glycoprotein (P-gp) expression at the intestinal epithelial barrier (68) and the BBB (69) but to reduce its activity (68, 69). P-gp is a protein highly expressed at the brain endothelium (70, 71), where it functions as an efflux transporter, and is involved in the clearance of Aβ across the BBB (70–72). In AD, P-gp expression and function at the BBB is decreased (66, 73–75), contributing to Aβ accumulation in the brain (74, 75). Interestingly, fecal supernatants isolated from AD patients decreased expression of P-gp in an intestinal epithelial monolayer culture, compared to supernatants from control subjects and elders with other dementia types (60). The expression of P-gp was influenced by the abundance of several gram-negative *Bacteroides* species. High levels of the species *B. dorei* increased P-gp expression while increased abundance of *B. fragilis* and *B. vulgatus* correlated to decreased expression of P-gp (60).

In addition, the presence of meso-diaminopimelic acid (meso-DAP) in the peptidoglycan layer of the bacterial cell wall, which is part of all gram-negative and some gram-positive bacteria, is recognized by the nucleotide-binding oligomerization domain-containing protein 1 (NOD1) (76). Excretion or translocation of gram-negative peptidoglycan increased activity of bone marrow-derived neutrophils *via* NOD1 receptor signaling (77).

#### Gram-Negative Bacteria: PSA Producers

Polysaccharide A (PSA) is a capsular carbohydrate that specifically derives from the gram-negative species *B. fragilis*. PSA from *B. fragilis* promotes regulatory immune responses *via* binding to TLR-2 (26–28), including the induction of dendritic cells (DCs) and regulatory T cells (Treg) (26–28) and the secretion of IL-10 (28, 78), together with subsequent inhibition of Th17 cells (26, 27) and suppression of IL-17 production (79). Colonization of germ-free (GF) mice with PSA-producing *B. fragilis* led to an expansion of CD4^+^ T cell levels in the spleen and restored the Th1/Th2 cytokine balance of GF mice by reducing IL-4 production and restoring interferon γ (IFN-γ) expression (80). Interestingly, another study that investigated PSA-mediated stimulation of CD4^+^ T cells found that cells responding to PSA displayed an unusual combination of pro-inflammatory cytokines (IFN-γ, TNF-α, IL-6 and C-X-C motif chemokine ligand 10) and anti-inflammatory surface receptor expression (Lag3, Tim3, Pd1) that was mainly driven *via* the interferon signaling pathway (81). Hence, the immunological response to PSA exposure is highly context dependent and cannot be considered simply regulatory or pro-inflammatory.

### Butyrate-Producing Bacteria

Short-chain fatty acids (SCFAs) promote gut integrity and play an important role in both physiological and pathological conditions (82–84). The most abundant SCFAs are acetate, butyrate and propionate, of which butyrate is particularly essential in the gut, as it is the most important metabolic substrate required for colonocyte proliferation and differentiation (82, 85). Butyrate-producing bacteria are widely distributed amongst the gram-positive phylum *Firmicutes* (16). Two of the most important butyrate-producing species are *F. prausnitzii* and *E. rectale* (29, 86), which exert different effects on peripheral immunity, including the induction of Treg cells (87, 88) and inhibition of NF-κB signaling in the intestinal epithelium (89). Additional butyrate producing taxa can be found within other bacterial families, mostly in *Lachnospiraceae* (see **Figures 2** and **3**) (90–98)

Butyrate, like other SCFAs, exerts its effects by acting as an histone deacetylase (HDAC) inhibitor (99) and *via* G-protein receptor (GPCR) signaling (29), which can both lead to inhibition of the NF-κB signaling pathway (100–103). Butyrate-mediated inhibition of HDAC signaling downregulated inflammatory mediators IL-6, IL-12 and nitric oxide synthase 2 (NOS2) in LPS-treated macrophages *in vivo*, and *in vitro* (31). Furthermore, butryate-induced GPCR signaling increased IL-10 expression in splenic dendritic cells (DCs) and macrophages *in vitro* (30) and enhanced plasma IL-10 levels *in vivo* (104). DCs and macrophages cultured with butyrate had increased potency to induce differentiation of Treg cells (30, 104–107) and promoted IL-10 production by CD4^+^ T cells, while decreasing levels of IL-17 (30). Butyrate-induced Treg differentiation *in vitro* was dependent on transforming growth factor β1 (TGF-β1) (107). Notably, butyrate administration in rats reduced IL-6, IL-17 and IL-23 levels and increased levels of TGF-β in the plasma (104). DCs exposed to butyrate could also suppress differentiation of naïve T cells into pro-inflammatory IFN-γ producing T cells (105). Butyrate also reduced the production of TNF-α and cytokine-induced neutrophil chemoattractant (CINC) 2αβ and nitric oxide (NO) in LPS-treated rat neutrophils *in vitro* (99).

However, a high dose of butyrate induced IFN-γ and T-bet expression, both associated with a Th1 phenotype, in CD4^+^ T cells cultured under Treg cell inducing conditions (107). Unlike the induction of Treg cells, induction of these Th1 associated factors was not dependent on TGF-β1. Under Th17-polarizing conditions, butyrate inhibited RORγt and IL-17A but induced IFN-γ, while under Th2-polarizing conditions, butyrate decreased expression of GATA3 and IL-4 and induced IFN-γ. The upregulation of IFN-γ under these conditions is dependent on the expression of T-bet. In addition, butyrate upregulated IFN-γ in a concentration dependent manner in unpolarized T cells (107). As such, it has been proposed that butyrate might exert its pro-inflammatory potential in an inflammatory context, while it shows anti-inflammatory effects under homeostatic conditions (107). Still, most evidence points to butyrate as a potent anti-inflammatory SCFA both *in vivo* and *in vitro*.

## Immune-AD Associations

While AD pathology was long considered to be driven mainly by Aβ and tau pathology, accumulating evidence shows that dysfunctional neuro-immunological responses considerably contribute to AD pathogenesis and might even be a driving factor (5). Rare genetic variants associated with AD are often highly expressed in microglia, including the triggering receptor expressed on myeloid cells 2 (TREM2) (108). Rare variants of TREM2 are associated with a two- to threefold increase in risk of AD development (109). Besides, the role of the immune system in AD is not limited to the brain, but also involves peripheral immune signaling (4, 5, 110). Accordingly, blood-derived leukocytes were identified in the brain of AD patients and AD animal models (111–113), and infiltration of these peripheral immune cells into the brain during disease pathogenesis can be facilitated by an increase in BBB inflammation and enhanced permeability (114).

Through the release of cytokines, complement proteins and major histocompatibility complex (MHC) class I proteins, peripheral immunity can affect CNS homeostasis (115). Besides, factors that exert their functions in the CNS, like neurotransmitters, are involved in the mediation of immune responses through corresponding receptors on innate and adaptive immune cells (116–118). In addition, the CNS can control systemic immune responses *via* the vagus nerve (119), and the sympathetic branch of the autonomic nervous system can influence intestinal immunity and homeostasis (120). Activation of the HPA axis and subsequent release of glucocorticoids also greatly affects immune responses (121). The recently discovered brain lymphatic system (122–124) and the regulation of immune cell trafficking across the BBB (125) further contribute to communication between the peripheral immune system and the CNS (125). As discussed, microbiota can exert different effects on central and peripheral immunity *via* their metabolites and toxins, thereby affecting central and peripheral inflammatory processes in AD.

## Therapeutic Strategies Targeting the Gut Microbiota and Metabolites in AD

### Pro-, Pre- and Antibiotics

#### Probiotics

Probiotics consist of living microbes and can introduce beneficial microbial components that are missing in the host (126). In APP/PS1 mice, treatment with probiotics containing *Bifidobacterium longum* and *Lactobacillus acidophilus* in combination with exercise was able to inhibit the progression of cognitive impairment and Aβ deposition (50). These species, among other strains within *Bifidobacterium* and *Lactobacillus*, provide cross-feeding to butyrate-producers (127). Before treatment, APP/PS1 mice showed higher abundance of several *Bacteroides* species and a reduction of butyrate-producing strains compared to WT mice. Probiotic treatment in combination with exercise decreased the gram-negative species *B. fragilis* and *Bacteroides thetaiotaomicron*, of which the latter was related to poorer spatial memory, while both of these species were increased by probiotics alone. Butyrate-producing genera like *Eubacterium* and *Roseburia* were enhanced by probiotic treatment in combination with exercise, but decreased by probiotic treatment alone. Exercise without probiotic supplementation was also able to reverse the alterations in butyrate producing species and in *B. fragilis* that were seen in APP/PS1 mice, but did not decrease *B. thetaiotaomicron*. Accordingly, spatial memory was improved by exercise and probiotic treatment combined, but not considerably altered by exercise or probiotics separately. Aβ pathology was decreased by probiotics and exercise separately, and by combined treatment (50).

AD patients who received probiotics containing species of *Bifidobacterium bifidum*, *Lactobacillus fermentum*, *Lactobacillus casei* and *Lactobacillus acidophilus*, which provide cross-feeding to butyrate-producing bacteria, showed improvement on the Mini Mental State Exam (MMSE) compared to untreated patients (128). Probiotic treatment in AD patients also resulted in favorable changes in insulin metabolism and in malondialdehyde (MDA) and high sensitivity C-reactive protein (hs-CRP), which are markers for oxidative stress and inflammation respectively, but was ineffective on other biomarkers of oxidative stress and inflammation such as total antioxidant capacity (TAC), nitric oxide (NO) and glutathione (GSH). Of note, the effect of probiotic treatment on microbiota composition was not investigated and as such, no firm conclusions can be drawn whether results were mediated by probiotic-induced changes in microbiota composition (128).

#### Prebiotics

Prebiotics are defined as substrates selectively used by host microorganisms to produce health benefits. The main source of prebiotics are plant-derived carbohydrate compounds called oligosaccharides (129). Prebiotics are non-digestible by the host, selectively fermented by intestinal microorganisms and selectively targeting and stimulating growth and activity of beneficial bacteria, especially *Bifidobacterium* and, to a lesser extent, *Lactobacillus* (129). Rats that received a hippocampal injection of Aβ42 and were orally treated with oligosaccharides from *Morinda officinalis* (OMO) afterwards show improved learning and memory in a dose dependent way, ameliorated neuronal loss, decreased Aβ42 expression and reduced oxidative stress. In these rats, OMO treatment decreased both pro-inflammatory cytokines and anti-inflammatory IL-10 to a level similar to WT. Moreover, OMO treatment restored the abundance of both *Bacteroidetes* and *Firmicutes* to WT levels (130). In APP/PS1 mice, OMO treatment induced an increase in *Firmicutes*, particularly in the butyrate-producing family *Lachnospiraceae*, while a decrease was seen in *Bacteroidetes* and the genus *Bacteroides*. Interestingly, an increase in *Firmicutes* and *Lachnospiraceae* and a reduction in *Bacteroidetes* were also observed in APP/PS1 mice compared to WT. Similar to rats, OMO treatment improved learning and memory in a dose dependent way (49). Together, these studies show that OMO treatment can affect different aspects of AD pathology like neuronal loss, cognitive deficits, inflammation, oxidative stress and Aβ42 expression, and that OMO might exert these effects *via* modulating microbiota composition (49, 130).

#### Antibiotics

Antibiotics are commonly used to limit bacterial colonization of the body, without targeting specific taxa, and can lead to significant alterations in gut microbiota composition (131).

Studies have reported that antibiotic treatment can ameliorate neuroinflammation and other aspects of AD pathology, including Aβ and tau accumulation and oxidative stress (132). Antibiotic treatment in AD mouse models affected AD pathology in a sex-specific manner. Male APP/PS1 mice showed a significant decrease in Aβ plaque compared to untreated animals after antibiotic treatment, a result that was not observed in females (133). In males, antibiotic treatment reduced microglial and astroglial reactivity around Aβ plaques (133), while an activated microglial phenotype was observed independent of antibiotic treatment in female mice (134). Additionally, antibiotic treated males had decreased expression of pro-inflammatory cytokines like IL-1β and IL-17A, while these cytokines were increased in antibiotic treated females. Furthermore, the antibiotic treatment induced sex-specific changes in microbiota composition. Also, antibiotics inhibited pathways related to LPS synthesis, but this effect was stronger in males than in females (134). These results were at least partially microbiome-dependent since microbiota transplantation from untreated male APP/PS1 mice to antibiotic treated mice resulted in partial restoration of Aβ deposition and microglial morphology (134).

So far, contradicting results have been reported between clinical trials. One study demonstrated that high doses (50-100 mg) of the antibiotic D-cycloserine, administered over a period of 4 weeks, improved cognition in AD patients (135), while an earlier study that treated patients with lower doses (15 mg) for the same period showed no effects (136). In 2004, a combined treatment of doxycycline and rifampicin for a period of 3 months resulted in significantly less cognitive decline over the 6-month period after the start of the treatment in patients with probable AD and mild to moderate dementia, compared to the placebo treated group (137). In contrast, a later study in 2013 found no beneficial effects of a 12-month treatment with either doxycycline or rifampin, or combined treatment, on cognition in AD patients (138).

The reason for these discrepancies regarding the effects of antibiotics might be the multifactorial nature of AD, or other systemic effects of the antibiotic besides changing microbiota composition. The outcome of clinical trials can also be affected by participants being infected with *H. pylori*, which is quite common in older patients. As such, cognitive improvement that is observed in infected patients might be a result of elimination of the *H. pylori* infection by antibiotics (131, 139). Also, while some studies examined the effect of antibiotic treatment directly after the treatment period (135, 136, 138), one performed cognitive assessment months after the treatment had stopped (137). In both cases, the treatment was reported to improve cognitive decline (135, 137), suggesting cognition was improved by a significant reduction in microbial diversity or in specific taxa right after antibiotic treatment, but also by a changed composition after repopulation. Still, other studies showed no effect of antibiotics directly after the treatment (136, 138).

In summary, research suggests that the use of antibiotics can at least interfere with AD pathology and associated neuroinflammation. However, microbiome-independent effects of antibiotics in GF mice, which lack microbiota, have been described that included changed host metabolites and inhibited respiratory activity in immune cells, consequently impairing immune phagocytic activity (140). In addition, antibiotic treatment has shown to induce FoxP3^+^ Treg cell in GF animals (141). Hence, it can be debated if the reported neuro-protective effects of antibiotic treatment were mediated *via* changes in the gut microbiome or *via* other pathways. Future research should elucidate if the effects of antibiotic treatment are mediated *via* changes in microbiota composition, *via* direct effects on immune cells or *via* other pathways in the host.

### Polysaccharide A (PSA) Treatment

No research has yet been performed on the possible beneficial effects of PSA on cognition or neuroinflammation in AD. However, PSA treatment reduced disease severity of mice with experimental autoimmune encephalomyelitis (EAE), which is often used to model the neuro-inflammatory disease multiple sclerosis (MS) (142). In addition, treatment of EAE mice with PSA reduced neuroinflammation by reducing pro-inflammatory cytokines in a TLR2 dependent manner (143) and through inhibition of Th1 and Th17 responses (142). If PSA administration can interfere with pathological processes in AD remains to be investigated. However, since the immunological response to PSA exposure could be dependent on the inflammatory context (81), other therapeutic strategies targeting the gut microbiota and metabolites in AD might be more promising for future research.

### Butyrate Treatment

Treatment with butyrate in GF male mice decreased BBB permeability and increased expression of the tight junction protein occludin. The same effect on BBB permeability was observed after monocolonization of GF mice with the butyrate producing bacteria *Clostridium tyrobutyricum* (144). Besides, pretreatment of adult and aged mice with a single injection of butyrate decreased LPS-induced IL-1β expression in microglia. Notably, the observed effect was stronger in aged mice. Pretreatment with butyrate also decreased LPS-induced IL-1β expression in the hippocampus of aged mice (145).

In APP/PS1 mice studies at advanced disease stage, treatment with butyrate resulted in improved memory function *via* HDAC inhibition, but did not affect Aβ pathology (146). Accordingly, treatment of aged Tg2576 mice with butyrate also improved cognition without affecting Aβ pathology, but decreased tau pathology and improved synaptic plasticity (147). In 5XFAD mice, butyrate both decreased Aβ deposition and improved cognition. Here, the effect of the treatment in an early disease stage was examined (148), suggesting that the effect of butyrate on Aβ deposition might be dependent on disease stage. Additionally, treatment of APP/PS1 mice with *Clostridium butyricum* increased fecal butyrate concentrations and ameliorated cognitive deficits and neurodegeneration, suppressed microglia activation and decreased levels of the pro-inflammatory cytokines Il-1β and TNF-α. Besides, *Clostridium butyricum* reversed microbiota alterations that were observed in APP/PS1 mice (149). No studies have yet been performed on the effect of butyrate treatment on AD pathology in humans. Altogether, usage of butyrate both reversed microbiota alterations and was able to interfere with neuroinflammation, BBB permeability, cognitive decline and, in early stage, pathological hallmarks like Aβ and tau in mouse models of AD.

### Additional Dietary Interventions

#### Calorie Restriction

The effects of dietary restriction, which can be either caloric reduction or intermittent fasting, on neuroinflammation are well summarized by Bok et al., which states that dietary restriction can reduce neuroinflammation *via* several mechanisms, including inhibition of the NF-κB pathway, or attenuation of aged-associated pro-inflammatory activation of astrocytes and microglia (150). Calorie restriction (CR) rescued most microbiota alterations that occur with increasing age in Tg2576 AD mice, and downregulated genes associated with intestinal inflammation (54) and reduced Aβ pathology (151). In contrast, CR also upregulated transcription factor Rorγt, which promotes a Th17 response (54). Interestingly, the CR diet as described by Cox et al. restricts only in carbohydrates (54), which are a source for butyrate production (16). However, if CR lowered butyrate levels was not investigated.

#### High Fiber Diet

Dietary intervention with fibers has shown to affect gut microbiota composition and levels of SCFAs. Supplementation of diets of healthy young adults for 2 weeks with three fermentable fibers resulted in an increase in SCFAs, including butyrate. As a response to resistant starch from potatoes, some participants showed an increase in *Ruminococcus bromii* or *Clostridium chartatabidum*, and this was associated with higher butyrate concentrations, especially in the presence of *E. rectale* (152). High fiber diet decreased the expression of inflammatory cytokines like IL-1β, TNF and IL-6 in microglia from adult and aged mice (145). Expression of these cytokines was negatively correlated to cecal levels of butyrate (145). Hence, a high fiber diet is able to modulate neuro-immunological processes probably *via* an increase in butyrate levels.

#### Mediterranean Diet

Adherence to a Mediterranean diet (MD) is associated with a lower risk for developing AD and delay in cognitive decline (153). This diet is characterized by high intake of fruits, vegetables, legumes, nuts, cereals, olive oil and fish, moderate intake of dairy, low intake of meat, and small quantities of wine (154). Through its antioxidant properties, MD is beneficial in combating oxidative stress in AD (153). Besides, dietary components like beneficial unsaturated fatty acids provide anti-inflammatory actions (155), and MD was found to be associated with lower levels of inflammatory markers like C-reactive protein (CRP) and IL-6 (156). MD also affects the gut microbiota and its metabolites, and has been linked to increased production of SCFAs (157). A cohort study with 153 Italian individuals following different diets showed that adherence to the MD correlates to higher fecal levels of acetate, propionate and butyrate (158). Accordingly, MD has been associated with increased abundance of butyrate-producing strains like *F. prausnitzii* (157, 159) and *E. rectale* (160) and the butyrate-producing genus *Roseburia* (161) and to reduced levels of circulating LPS (162).

### Fecal Microbiota Transplantation

Fecal microbiota transplantation (FMT) is the infusion of feces from a healthy donor into the gut of a recipient with the aim of targeting microbiota composition and is a promising strategy for combating disease associated with microbiota imbalance (163–168). FMT treatment is generally considered safe, especially after extensive donor screening and testing (169, 170) but remains technically challenging (donor selection and preparation of the fecal transplant). In the future, specific supplementation of a (combination of) beneficial strains could be more feasible on a larger scale (169).

In APP/PS1 mice, FMT from WT mice for a period of 4 weeks improved spatial memory and reduced Aβ accumulation and tau phosphorylation. Besides, FMT increased expression of proteins involved in synaptic plasticity, PSD-95 and synapsin I, and decreased inflammatory protein Cox-2 in the cortex and hippocampus. In addition, FMT reduced CD11b expression, which is a marker for microglia and other myeloid cells (171, 172), that was increased in APP/PS1 compared to WT mice. Alterations in microbiota composition in APP/PS1 mice compared to WT, including a reduction in *Bacteroidetes*, were reversed by FMT. Moreover, FMT increased levels of butyrate (171). Another study shows that FMT from WT to ADLPAPT mice, a relatively newly developed AD mouse model, resulted in decreased formation of Aβ plaques and neurofibrillary tangles, reduced glial activity and improved cognition (173). So far, no studies have investigated the effect of FMT in AD patients. However, an effect of FMT on the brain was demonstrated in obese patients, where FMT increased dopamine transporter binding which was associated with an concomitant increase in *Bacteroides uniformis* (174).

Overall, it seems that FMT treatment in AD mouse models has the potential to reverse microbiota alterations, improve cognition and synaptic plasticity, decrease Aβ and tau pathology and to reduce neuroinflammation (171, 173).

## Discussion

This review shows different alterations in microbiota composition in AD. Compositional differences across AD mouse studies were contradicting, as gram-negative and butyrate-producing bacteria were both increased and decreased in abundance. Limited but available human data revealed a higher abundance of gram-negative species within *Bacteroidetes* while species within *Firmicutes* that produce the metabolite butyrate were decreased. As such, it seems that results observed in mouse models of AD are not completely translatable towards humans.

Differences in microbiota composition between AD patients and AD animal models might be explained by differences in anatomy (175, 176) or in the used research techniques; while human studies use stool samples, cecal contents are mostly used in mouse studies (176). Another possible explanation is the absence of correction for confounding factors such as age, sex, diet, comorbidities, use of medication and inclusion of small sample sizes in human studies. Moreover, discrepancy in the findings might also be a result of the male/female ratio in these studies as sex-differences in gut microbiota composition have been acknowledged in humans (177) and in mice (56, 57, 178, 179). In AD mouse models, disease manifestation differs between males and females. Rapid and more severe AD pathology has been reported in females compared to males, including increased Aβ pathology (180, 181), tau phosphorylation (180) and neuroinflammation (181, 182) and cognitive deficits (183). Possibly, different disease manifestation between sexes in AD transgenic mouse models influences microbiota composition or vice versa. In humans, variation in AD disease manifestation has also been observed between men and women, and includes differences in cognitive symptoms and brain atrophy (184). Further research should shed more light on the possible relationship between sex-differences in disease manifestation and variations in microbiota composition in AD.

Lastly, all but one mouse study used a transgenic AD mouse-model with mutations associated with familial AD leading to early and excessive Aβ pathology. As such, detected shifts in microbiota composition are probably more characteristic to Aβ pathology of familial AD and can differ when other AD-associated pathologies are investigated. Also, mice and humans standardly show differences in microbiota composition, and raising the animals under specific pathogen free (SPF) conditions can reduce microbial diversity. Differences between mice and humans in microbiome research might be closed by using mice with humanized microbiota (185). However, generation of such animals is difficult, as successful transplantation of human gut microbiota into mice is under influence of anatomical and physiological factors, diet and environmental stimuli (186).

Innate immune activation in AD might differently affect pathogenesis dependent on disease stage. An activated microglial response could limit Aβ pathology in early or middle stages of AD (187–189), while it exacerbates tau pathology and neuronal loss in late stages (189, 190). Since the effects of immune activation on AD pathogenesis seem to be dependent on disease stage, the relation between the gut microbiome and AD could be as well. Hypothesizing, inflammatory effects of the gut microbiota might inhibit aspects of AD pathology in early disease stages, while they exacerbate pathology in late stages. The variation in microbiota composition in AD observed between different studies in humans (58–60) and animal models (44–55) might also be dependent on disease stage. Hence, it would be interesting to investigate functional changes in microbiota composition over the course of disease progression in humans together with shifts in immune status. Accordingly, animal studies show microbiota alterations in AD mouse models over time (48, 53, 54), suggesting that microbial changes correspond with disease progression.

Another possible way in which gut microbiota can induce immune activation is *via* their release of bacterial amyloids. Bacteria produce amyloids as part of their biofilm, a self-produced extracellular matrix which protects the population from different environmental and host insults (191). Although bacterial-derived amyloids differ in structure from CNS amyloid, it has been suggested that due to existing similarities in their tertiary structure (192, 193), bacterial amyloids may prime the immune system and induce misfolding of other host proteins through molecular mimicry (191, 194–197). For example, bacterial amyloid curli is recognized by the TLR2/TLR1 complex that also recognizes human Aβ. Apart from *Escherichia coli* and *Salmonella enterica serovar Typhimurium*, most of these observations are made *in vitro* (191). Hence, future research should reveal *in vivo* interactions between bacterial amyloid, the immune system and AD pathology.

**Figure 5:**
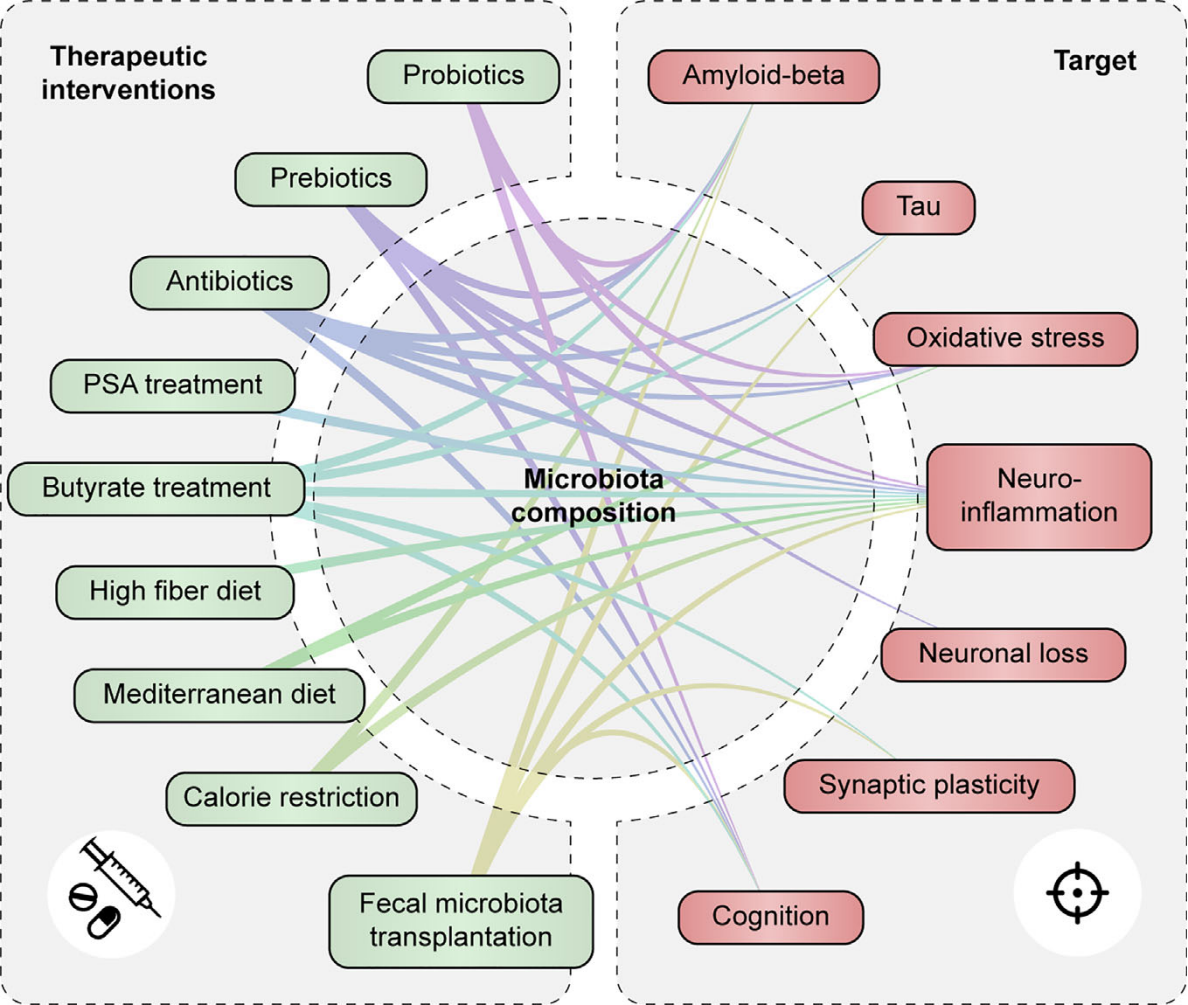
Therapeutic strategies targeting the gut microbiota and metabolites modulate AD-associated pathology. Associations are shown between therapeutic interventions that modulate gut microbiota composition and/or function and AD-associated pathologies such as tau and Aβ accumulation, oxidative stress and neuroinflammation, neuronal loss, synaptic plasticity and cognitive function.

Treatment strategies that target the microbiota and their metabolites, including pro-, pre- and antibiotics, butyrate treatment, and dietary interventions, have shown potential to modulate neuroinflammation and/or improve other aspects of AD pathology (**Figure 5** and **Supplementary Table 4**). Prebiotics and dietary interventions such as a high fiber might be able to restore the functional core of the microbiome by providing necessary dietary compounds for beneficial microbial metabolism. Less is known about butyrate-treatment or FMT as a therapeutic option for AD. However, both treatments were able to restore microbiota imbalance, enhance cognition and synaptic plasticity, decrease AD pathology and to reduce neuroinflammation in mouse models of AD. As such, these therapeutic options could be promising to follow up in future studies.

In conclusion, gut microbiota composition shows many changes in AD patients and animal models, despite some inconsistencies in compositional differences between studies. It is clear however, that species of bacteria affect central and peripheral immune networks and have the ability to modulate ongoing neuro-immunological responses. Thus, restoring misbalanced microbiota in AD may present a future measure to increase immune fitness and alleviate AD pathology.

Still, recovering microbial balance with no general consensus on what characterizes the AD-associated microbiome is doomed to face long odds. To overcome inconsistencies between studies in this field, future research should move towards studying the functionality and dynamics of the human core microbiome in AD rather than static abundance of microbial taxa, for example, by studying levels of immunomodulating metabolites, like PSA or butyrate, in relation to AD. Also, a better understanding is needed of how such dynamic alterations affect immune pathways and how these pathways can be therapeutically targeted. A good starting point would be the use of shotgun sequencing (38, 198, 199) accompanied by immune profiling at different time-points of disease. Larger sample sizes in clinically well characterized cohorts could enable assessment of confounding/mediating effects of sex and other host factors such as diet in the relation between microbiota function, immune status and AD pathology. Finally, future studies should address how the microbiota-associated changes relate to AD biomarkers and disease severity, such as cerebral spinal fluid (CSF) or PET biomarkers for Aβ and tau, MRI characteristics or psychological assessment of cognitive functioning.

## Author Contributions

LO and SR wrote the manuscript. AK designed the figures. BV, AG, and MM provided valuable scientific input and revised the manuscript. LO, WF, and HV conceived the study and were involved in the overall supervision and editing of the manuscript. All authors contributed to the article and approved the submitted version.

## Funding

This work was financed by Horizon 2020 #686009 to HV.

## Conflict of Interest

The authors declare that the research was conducted in the absence of any commercial or financial relationships that could be construed as a potential conflict of interest.
