# Supplementary material for: Contribution of Gut Microbiota to Immunological Changes in Alzheimer’s Disease
Source: Front Immunol. 2021 May 31;12:683068. doi: 10.3389/fimmu.2021.683068 (PMC8200826; doi:10.3389/fimmu.2021.683068)
Supplement: Supplementary file 1 [file DataSheet_1.pdf]

Supplementary Material

Supplementary table 1

	AD model	WT model	Male/ Female	Age (months)	N (Tg/WT)		Microbiome comparison by	Known housing conditions	Diet	Sequencing method
Brandscheid et al, (2017) [44]	5XFAD	C57BL//6	M	1.5, 2, 4	≥18/18	≥6 per timepoint per groep	Genotype	12/12h light dark cycle	Unlimited food	16S rRNA amplicon sequcing
Harach et al. (2017) [45]	APP/PS1	C57BL/6	M + F	8	6/7		Genotype	SPF conditions, grouped	Unlimited food	16S rRNA amplicon sequcing
Shen et al. (2017) [46]	APP/PS1	C57BL/6	M	3, 6, 8	6/6 (at each age)		Genotype, age	SPF conditions, room temperature, 12/12h light-dark circle, caged	Unlimited food	16S rRNA amplicon sequcing
Zhang et al. (2017) [47]	APP/PS1	C57BL/6	M	1,3, 5-6, 8-12	24/24	6 per timepoint per group	Genotype	SPF conditions, controlled environmental	Unlimited food	16S rRNA amplicon sequcing
Bäuerl et al. (2018) [48]	APP/PS1	C57BL/6	F	3, 6, 24	8/9	(3,3,2 / 3,3,3 at timepoints)	Genotype, age	SPF conditions, room temperature, 12/12h light-dark cycle	Unlimited food	16S rRNA amplicon sequcing
Xin et al. (2018) [49]	APP/PS1	C57BL/6	M	2	10/10		Genotype, OMO treatment	25°C, 12/12h light-dark cycle, housed in pairs	Unlimited food	16S rRNA amplicon sequcing
Abraham et al. (2019) [50]	APP/PS1	C57BL/6	M	8	8/unknown		Genotype, probiotic + exercise treatment	Unknown	Unlimited food	Ion PGM Sequencing
Sun et al. (2019) [51]	P301L	FVB/N	M + F	3, 6, 10	32/32	(8 per timepoint)	Genotype	SPF conditions, 12/12h light-dark circle	Unlimited food	16S rRNA amplicon sequcing
Wang et al. (2019) [52]	APP/PS1	C57BL/6	M	9	6/6		Genotype, JAT treatment	Air-conditioned room, >12 hours of night in light-dark cycle	Unlimited food	16S rRNA amplicon sequcing
Wang, X. et al. (2019) [53]	5XFAD	C57BL/6	M + F	2-9	4-10/4-10 (at different ages)		Genotype (at 7 months), age	Room temperature, 12/12h light-dark cycle	Differs over course of experiment, food is limited before behavioral experiments	16S rRNA amplicon sequcing
Cox et al. (2019) [54]	Tg2576	Swiss Webster DBA/C57BL/6 F1	M + F	5-15 (mice are followed with increasing age)	15-17/15-17 (at the starting point)		Genotype, age, seks, CR diet	Temperature controlled conditions, 12/12h light-dark cycle	Unlimited food (Animals on CR diet are not included here)	16S rRNA amplicon sequcing
Chen et al. (2020) [55]	APP/PS1	C57BL/6	M	1,2,3,6,9	121/97	(21,24,24,34,18 / 14,17,17,31,18 at timepoints)	Genotype	SPF conditions, 24°C, 12/12h light-dark cycle, caged per 4 (WT and Tg together)	Unlimited food	16S rRNA amplicon sequcing

Supplementary table 1: Characteristics of animal studies regarding microbiota composition in AD mouse models compared to WT. The used AD mouse model and corresponding WT model is listed together with male/female ratio, age of the animals, sample size, reported comparison, known housing conditions, diet and the used sequencing technique per study.

Supplementary table 2

	Increased in AD mouse models	Decreased in AD mouse models
Phyla	Firmicutes[44](2) [48*, 49, 52, 53*]	Bacteroidetes[44](2) [48* 49, 52, 53*]
	Bacteroidetes[45, 51, 54]*(F)	Firmicutes[45, 51, 54]*(F)
	Deferribacteres[49, 52]	Verrucomicrobia[45, 52, 53*]
	Tenericutes[45]	Actinobacteria[45,51]
	Proteobacteria [47](5) [48](6) [55](9)	Tenericutes[51](10) [52]
	Verrucomicrobia [47](8) [55](2,6,9)	Proteobacteria[45]
	Actinobacteria (2)[55]	Cyanobacteria[49]
Families	Lachnospiraceae[49, 52]	Erysipelotrichaceae [49,52]
	Deferribacteraceae[49, 52]	Prevotellaceae[49, 52]
	Helicobacteriaceae[46, 52]	Coriobacteriaceae[49, 51](10)
	Desulfovibrionaceae[46, 49, 55](9)	Bifidobacteriaceae[49, 51](6)
	Bacteroidaceae[49, 51](6) [54]*(F)	Pepto-streptococcaceae[52]
	Rikenellaceae[49, 54]*(F)	Bacteroidales_S24_7_group[52]
	Prevotellaceae[46*] [51](10) [55](2)	S24-7[49]
	Verrucomicrobiaceae[49, 55](2,6,9)	Ruminococcaceae[49]
	Lactobacillaceae[49]	Enterobacteriacea[49]
	ClostridialesvadinBB60group[51](6,10) [54]*(F) [55](9)	Clostridiaceae[49]
	Erysipelotrichaceae[48](24) [55](2)	Helicobacteriaceae[49]
	Marinifilaceae[54]*(F)	[Paraprevotellaceae] [49]
	Tannerellaceae[54]*(F)	Alcaligenaceae[49]
	Coriobacteriaceae[46](6,8)	Lachnospiraceae[51](3) [54]*(F)
	Bacteroidales_S24_7_group [51](3,10)	Streptococcaceae[51](10) [54]*(F)
	Acidaminococcaceae[51](3)	Staphylococcaceae[51](3,6) [54]*(F)
	Alcaligenaceae[51](3)	Rikenellaceae[48](24) [55](6)
	Unclassified_o_Bacteroidales[51](3,10)	Carnobacteriaceae[51](3) [55](9)
	Anaeroplasmataceae[51](6)	Lactobacillaceae[51](3)
	Porphyromonadaceae[51](10)	Aerococcaceae[51](3)
	Enterobacteriaceae[55](1,6)	Desulfovibrionaceae[51](3)
	Bifidobacteriaceae[55](2)	Vibrionaceae[51](3)
		Fusobacteriaceae[51](3)
		Family_XIII[51](3)
		Clostridiaceae_1[51](3)
		Actinomycetaceae[51](3)
		Family_XI_o_Bacillales[51](6)

		Mycoplasmataceae[51](10)
		Bacteroidaceae[55](6)
Genera	Helicobacter[46, 52, 53]	Allobaculum[45, 52]
	Bacteroides[49, 50, 51](6) [54]*(F)	Akkermansia[45, 52]
	Odoribacter[46, 51, 54]*(F)	Prevotella[46, 49]
	Desulfovibrio[49, 53, 55](9)	Alloprevotella[52, 53]
	Mucispirillum[49, 52]	RuminococcaceaeUCG_0144[52, 53]
	Roseburia[53, 54]*(M)	Clostridium[49, 50]
	Akkermansia[49, 55](2,6,9)	Ruminococcus[46](6,8) [47] (8) [49]
	Ruminoclostridium[51](10)	Roseburia[50, 51](10)
	Norank_f_Ruminococcaceae[51](10) [53]	Bifidobacterium[49, 51](6)
	Lactobacillus[49]	Bacteroides[53, 55](6)
	Adlercreutzia[49]	Lactobacillus[51](3) [53]
	Lactococcus[53]	Alistipes[53, 55](9)
	Eubacterium_xylanophilum_group[53]	Eubacterium_brachy_group[51](3) [52]
	Ruminoclostridium_9[53]	Rikenellaceae_RC9_gutgroup[53, 55](6)
	Lachnospiraceae_NK4A136_group[53]	Oscillospira[49]
	Unclassified_f_lachnospiraceae[53]	Helicobacter[49]
	Norank_f_lachnospiraceae[53]	Anaerostipes[49]
	Candidatus_Saccharimonus[53]	Blautia[49]
	Parabacteroides[51](3) [54]*(F)	Eubacterium[50]
	Alloprevotella[54]*(M) [55](2)	Staphylococcus[51](3,6) [54]*(F)
	RikenellaceaeRC9gutgroup[54]*(F)	Turicibacter[54]*(F/M)
	MuribaculaceaeOther[54]*(F)	LachnospiraceaeUCG_006[54]*(F/M)
	Christensenellaceae Uncult[54]*(F)	Faecalibaculum[54]*(F)
	ClostridialesvadinBB60group[54]*(F)	Lactococcus[54]*(F)
	Negativbacillus[54]*(F)	Sporosarcina[54]*(F)
	Streptococcus[54]*(M)	PrevotellaceaeUCG_001[54]*(M)
	Eubacterium_coprostanoligenesgroup[54]*(M)	Mucispirillum[54]*(M)
	Norank_f_Bacteroidales_S24_7_group[51](3,10)	Butyricicoccus[47](8) [51](3)
	Unclassified_o_Bacteroidales[51](3,10)	Enterorhabdus[51](3)
	Ruminococcaceae_NK4A214_group[51](6)	Aerococcus[51](3)
	Norank_f_Clostridiales_vadinBB60_group[51](6)	Desulfovibrio[51](3)
	Anaerovorax[51](10)	Ignatzschineria[51](3)
	Peptococcus[51](10)	unclassified_f_Lachnospiraceae[51](3)
	Caproiciproducens[51](10)	Lachnospiraceae_FCS020_group[51](3)
	Oscillibacter[51](10)	Jeotgalicoccus[51](3)
	PrevotellaceaeUCG_001[55](2)	Vibrio[51](3)
	Bifidobacterium[55](2)	Psychrilyobacter[51](3)

	Allobaculum[55](2)	Ruminococcus_2[51](3)
	Erysipelatoclostridium[55](2)	Butyrivibrio[51](3)
	LachnospiraceaeUCG_001[55](3,9)	Ruminococcacaea_UCG_0044[51](3)
	Blautia[55](6)	Unclassified_f_Erysipelotriachaceae[51](3)
	Escherichia/Shigella[55](6)	Granulicatella[51](3)
	Tyzzerella[55](6)	Marvinbryanta[51](3)
	Family_XIII_UCG_001[55](6)	Actinomyces[51](3)
	Turicibacter[55](6)	Lachnoclostridium[51](3,10)
	Uncultered_bacterium_f_clostridiales_vadinBB60_group[55](9)	unclassified_f_Coriobacteriaceae[51](3,10)
	Ruminococcus_1[55](9)	Norank_f_mycoplasmataceae[51](10)
		Klebsiella[51](10)
		Streptococcus[51](10)
		Ruminoclostridium_549[5](3)
		RuminococcaceaeUCG_010[55](3)
		[Eubacterium]_nodatum_group[55](9)
		Atopostipes[55](9)
Species	L acidophilus[52]	B. proteoclasticus[50]
	B. fragilis[50]	L. johnsoni[50]
	B. thetaiotaomicron[50]	B. pullicaecorum[47](8)
	C. leptum[44](2)	
	Desulfovibrio C21_C20[47](5)	

Supplementary table 2: Microbial taxa are altered in AD mouse models compared to WT mice. Animal studies that either compare microbiota composition between AD and WT mice at certain time point(s), or that examine alterations with increasing age in AD compared to WT mice. * indicates a result was observed in AD with increasing age, but not in WT mice. Numbers behind taxa as () indicate this result was only observed at these time points. F or M show a change was only seen in females or males respectively, if both were included in one study.

Supplementary table 3

	Vogt *et al.* (2017)[58]		Zhuang *et al.* (2018)[59]		Haran *et al.* (2019)[60]		Cattaneo *et al.* (2017)[61]		
	**AD**	**Control**	**AD**	**Control**	**AD**	**Control**	**Patients Aβ negative**	**Patients Aβ postive**	**Control Aβ negative**
N	25	25	43	43	24	51	33	40	10
**age, y (mean ± SD)**	71.3 ± 7.3	69.3 ± 7.5	70.1 ± 8.8	69.7 ± 9.2	84.7 ± 8.1	83.0 ± 10.2	71 ± 7	70 ± 7	68 ± 8
**Sex (% Female)**	72%	68%	46.50%	46.50%	83.30%	84.30%	83.30%	55.55%	60.00%
**APOE ε4 genotype (%)**	72%	20%	39.50%	23.30%	*Unknown*	*Unknown*	*Unknown*	*Unknown*	*Unknown*
**Ethnicity**	Caucasian (96%)	Caucasian (92%)	Chinese	Chinese	*Unknown*	*Unknown*	*Unknown*	*Unknown*	*Unknown*
**AD diagnosis**	Clinical diagnosis of 'dementia due to AD'		Clinical diagnosis		Clinical diagnosis (confirmed by facility medical record and treating physician)		No AD diagnosis, Aβ positivity was based on Amyloid PET, cognitive impairment through clinical assessment including Mini-Mental State Examination		
**Sequencing method**	16S rRNA amplicon sequencing		16S rRNA amplicon sequencing		Shotgun metagenomic sequencing		Microbial DNA qPCR Assay Kit		

**Patients recruited from**	Wisconsin Alzheimer's Disease Research Center (ADRC) & Wisconsin Registry for Alzheimer's Prevention (WRAP)	Daping Hospital, Southwest Hospital of Third Military Medical University, First Affiliated Hospital of Chongqing Medical University & Chongqing People's Hospital (China)	4 nursing home (NH) facilities in central Massachusetts, approved by University of Massachusetts Medical School	A larger study in 18 memory clinics in Eastern Lombardy, Italy, aiming to assess the added value of amyloid imaging in the clinical work-up of patients with cognitive complaints (the Incremental Diagnostic Value of Florbetapir Amyloid Imaging [INDIA-FBP] study)

Supplementary table 3: Characteristics of human studies regarding microbiota composition in AD patients compared to aged-matched control subjects. Sample size of the studies is listed, together with mean age of the subjects, male/female ratio, % APOEε4 genotype, ethnicity, AD diagnosis of the included patients, the sequencing method used and facilities where the patients were recruited from.

Supplementary table 4

Therapeutic Intervention	Gut microbiota compostion	Metabolite levels	Effects on AD pathology						
			(Neuro)inflammation	Aβ pathology	Tau pathology	Cognition	Neuronal loss	Synaptic plasticity	Oxidative stress
Probiotics	↑ *B. longum* (50[A])<br>↑ *B. bifidum* (128[H])<br>↑ *Prevotella* spp. (50[A])<br>↑ *Bacteroides* spp. (50[A])<br>↑ Lactobacillus spp. (50[A], 128[A])<br><br>↓ *Eubacterium* spp. (50[A])<br>↓ *Roseburia* spp. (50[A])<br>↓ *Clostridium* spp. (50[A])<br>↓ *B. proteoclasticus* (50[A])<br>↓ *M. formatexigens* (50[A])<br>↓ *L. jonhsonii* (50[A])	↑ Butyrate (via crossfeeding (50[A], 128[H])	↓ (128[H])	↓ (50[A])		↑ (128[H])			↓ (128)[H]
Probiotics + exercise	↑ *L. acidophilus* (50[A])<br>↑ *B. longum* (50[A])<br>↑ *Eubacterium* spp. (50[A])<br>↑ *Roseburia* spp. (50[A])<br>↑ Lactobacillus spp. (50[A])<br>↑ *B. proteoclasticus* (50[A])<br>↑ *M. formatexigens* (50[A])<br><br>↓ *Prevotella* spp. (50[A])<br>↓ *Bacteroides* spp. (50[A])<br>↓ *Clostridium* spp. (50[A])<br>↓ *L. jonhsonii* (50[A])	↑ Butyrate (via crossfeeding (50[A])		↓ (50[A])		↑ (50[A])			
Prebiotics	↑ *Bifidobacterium* (129[H])<br>↑ *Bacteroidetes* (130[A])<br>↑ *Firmicutes* (130[A])<br>↑ *Lactobacillus* (130[A])<br><br>*Induces many alterations at phylum, family and genus level (49[A]), including:<br>↑ *Firmicutes*<br>↑ *Lachnospiraceae*<br>↑ *Lactobacillus*<br><br>↓ *Bacteroidetes*<br>↓ *Bacteroides*	↑ Butyrate (via increase in butyrate producers and crossfeeding) (49[A], 129[H], 130[A])	↓ (130[A])	↓ (130[A])		↑ (130[A]) (49[A])	↓ (130[A])		↓ (130[A])
Antibiotics	*Sex-specific changes, including alterations within *Bacteroidetes* and *Firmicutes* (134[A])	↓ LPS (134[A])	↓ (132[M], 133[A (M)])<br>↑ (134[A (F)])	↓ (132[A], 133[A (M)])	↓ (132[A])	↑ (135[H], 137[H])			↓ (132[A])
PSA treatment		↑ PSA (142[A], 143[A])	↓ (142[A], 143[A])						
Butyrate treatment	↑ *C. tyrobutyricum* (145[A])<br>↑ *C. butyricum* (149[A])<br>↑ *Alloprevotella* (149[A])<br>↑ S24-7 (149[A])	↑ Butyrate (144-149[A])	↓ (145[A], 149[A])	↓ (148[A])	↓ (147[A])	↑ (146[A], 147[A], 148[A], 149[A])	↓ (149[A])	↑ (147[A])	

	↓ *Deferribacteres* (149[A])<br>↓ *Helicobacteraceae* (149[A])<br>↓ *Helicobacter* (149[A])									
Calorie restriction	*rescues age-related alterations (54[A (F)])<br>Effects include:<br>↑ *Firmicutes*<br>↓ *Bacteroides*	↓ Butyrate (via restriction in carbohydrates (54[A])	↓ (150[A])	↓ (151[A])						
High fiber diet	↑ *R. bromii* (152[H])<br>↑ *Clostridium* spp. (152[H])<br>↑ *Bifidobacterium* spp. (152[H])<br>↑ *A. hadrus* (152[H])<br><br>↓ *Ruminococcus* spp. (145[A])<br>↓ *Rikenellaceae* (145[A])	↑ Acetate (145[A], 152[H])<br>↑ Butyrate (145[A], 152[H])	↓ (145[A])							
Mediterranean diet	↑ *F. prausnitzii* (157[H], 159[H], 160[H])<br>↑ *E. eligens* (157[H])<br>↑ *B. cellulosilyticus* (157[H])<br>↑ *Lachnospiraceae* (159[H])<br>↑ *Eubacterium* spp. (160[H])<br>↑ *Roseburia* spp. (159[H])<br>↑ *R. hominis* (160[H])<br>↑ *B. thetaiotaomicron* (160[H])<br>↑ *P. copri* (160[H])<br>↑ *A. hadrus* (160[H])<br><br>↓ *Clostridium* spp. (157[H])<br>↓ *C. aerofaciens* (157[H], 160[H])<br>↓ *Ruminococcus* spp. (157[H], 159[H])<br>↓ *R. lactatiformans* (159[H])<br>↓ *P. merdae* (159[H])<br>↓ *S. thermophilus* (159[H])<br>↓ *R. torques* (160[H])<br>↓ *F. plautii* (159[H], 160[H])<br>↓ *C. comes* (160[H])<br>↓ *D. formicigenerans* (160[H])<br>↓ *C. ramosum* (160[H])<br>↓ *V. dispar* (160[H])<br>↓ *A. lingnae* (160[H])<br><br>*many effects on microbiota composition described by 161[H], including:	↑ Acetate (158[H])<br>↑ Propionate (158[H])<br>↑ Butyrate (158[H])<br>↓ serum LPS (162[H])	↓ (155[H], 156[H], 160[H])			↑ (153[H], 160[H])			↓ (153[H], 155[H])

	↑ *Roseburia*								
Fecal microbiota transplantation	*Induces many alterations at phylum, family and genus level (171[A]), including:<br>↑ *Bacteroidetes*<br>↑ *Erysipelotrichia*<br>↑ *Faecalibaculum*	↑ Butyrate (171[A])	↓ (171[A], 173[A])	↓ (171[A], 173[A])	↓ (171[A], 173[A])	↑ (171[A], 173[A])		↑ (171[A])	

Supplementary table 4: The effects of microbiome-targeting therapeutic interventions on microbiota composition and metabolite levels, and on different aspects of AD pathology. ↑ indicates improvements in cognition or synaptic plasticity, while ↓ indicates a decrease in neuroinflammation, Aβ or tau pathology, neuronal loss or oxidative stress. "A" or "H" behind a reference demonstrates the results are obtained in animal or human studies respectively, while (F) or (M) shows a result was observed in female or male mice only. "Spp." behind a genus indicates that several species within this genus were altered. Effects written in grey are anticipated, but not actually measured.
